# Dual-Stream Spatiotemporal Networks with Feature Sharing for Monitoring Animals in the Home Cage

**DOI:** 10.3390/s23239532

**Published:** 2023-11-30

**Authors:** Ezechukwu Israel Nwokedi, Rasneer Sonia Bains, Luc Bidaut, Xujiong Ye, Sara Wells, James M. Brown

**Affiliations:** 1 School of Computer Science, College of Science, University of Lincoln, Brayford Pool, Lincoln LN6 7TS, UKjamesbrown@lincoln.ac.uk (J.M.B.); 2 Mary Lyon Centre at MRC Harwell, Oxfordshire OX11 0RD, UK; 3 Independent Researcher, Lincoln LN6 7TS, UK

**Keywords:** mouse phenotyping, machine learning, supervised learning, video classification, spatiotemporal

## Abstract

This paper presents a spatiotemporal deep learning approach for mouse behavioral classification in the home-cage. Using a series of dual-stream architectures with assorted modifications for optimal performance, we introduce a novel *feature sharing* approach that jointly processes the streams at regular intervals throughout the network. The dataset in focus is an annotated, publicly available dataset of a singly-housed mouse. We achieved even better classification accuracy by ensembling the best performing models; an Inception-based network and an attention-based network, both of which utilize this *feature sharing* attribute. Furthermore, we demonstrate through ablation studies that for all models, the *feature sharing* architectures consistently outperform the conventional dual-stream having standalone streams. In particular, the inception-based architectures showed higher *feature sharing* gains with their increase in accuracy anywhere between 6.59% and 15.19%. The best-performing models were also further evaluated on other mouse behavioral datasets.

## 1. Introduction

Over many decades, the ethical implications of using animals in research have undergone considerable discussion and scrutiny [1]. A major landmark in the regulation of research involving animals was the designation of the three ‘R’s (Replacement, Refinement, and Reduction), which was spearheaded by The National Centre for the 3Rs (NC3Rs) in the United Kingdom. As of 2022, around 2.76 million living animals were used for various research procedures in the UK, with 96% of these comprised of rodents (rats and mice), birds, and fish [2]. Due to their genetic, physiological, and anatomical similarities with humans [3] as well as their short lifecycles [4,5], mice are one of the most utilized species in biomedical research.

In support of the 3Rs mission, technology has been increasingly used to better understand the different aspects of research involving animals. Behavioral phenotyping is particularly important as it may highlight welfare concerns that arise over the course of an experimental design. However, the manual observation of these behaviors is expensive, laborious, and time-consuming. Furthermore, behavioral studies relying solely on expert observation are not easily reproducible [6,7]. The development of home-cage monitoring (HCM) systems was a major technological breakthrough that has helped to solve many of these issues [8]. HCM systems facilitate non-intrusive, longitudinal observation of mice and may provide a range of outputs such as behavioral annotation, ethogramming, depth sensing and tracking, activity summarising of circadian rhythm, and pose estimation. Such HCM systems include the Techniplast Digital Ventilated Cage (DVC) [9], the System for Continuous Observation of Rodents in Home-cage Environment (SCORHE) [10] and IntelliCage [11], to name a few. Cameras are extensively utilized across diverse industries for a number of tasks [12], including autonomous driving, pose estimation [13], security and surveillance, etc. As such, these home-cage setups may be equipped with either single-view [14] or multi-view cameras [10], depending on design considerations. Nevertheless, there are few commercially available solutions to the problem of detecting behaviors from video footage alone. Moreover, many of the solutions that do exist are strongly coupled to commercial hardware, rather than video footage in general. Owing to their recent successes in human action recognition and many other domains, deep learning approaches offer a potential solution to the problem of behavioural phenotyping in the home-cage.

**Figure 1:**
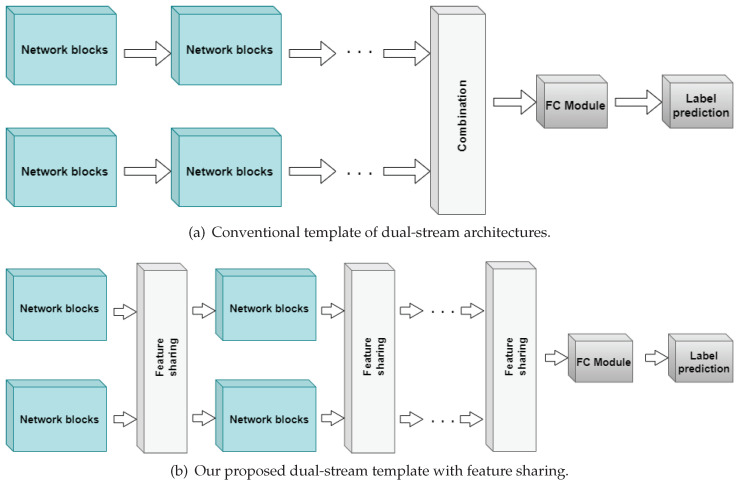
Conventional standalone vs. *feature sharing* dual networks. While the conventional dual-stream only extracts features for its stream, we propose the use of joint-processing layers, which we have termed *feature sharing*.

In this paper, dual-stream deep learning architectures are proposed for the behavioral classification of mice in the home-cage. The models in question were developed for entirely supervised learning, whereby spatiotemporal (ST) blocks of video data are mapped to one of several behavior categories. The dataset utilized is publicly available and contains videos of a singly-housed mouse [7]. Our models are initially trained on the entire main data and then tested using the more unambiguous clipped database. This approach is different compared to that in the original paper. Nevertheless, comparisons were also made between our proposed methodology and their results [7] using the same cross-validation technique adopted in the original publication. Furthermore, a select few of our models were also evaluated on a more complex, multi-view home-cage data [10]. One of the novel aspects of these models is shared layers between the streams of the networks. Here, instead of fusing individual streams at the end (termed “late fusion” in [15], we propose to combine features at regular intervals throughout the architecture. We hypothesize that accurate representations are better enforced when both streams are privy to information from each other (Figure 1). Some instances of shared features have been seen in U-Nets [16] and its many derivative networks, and some other specialized multi-stream architectures [17,18], albeit in a different manner to that proposed herein for multi-stream networks.

To the best of our knowledge, our work is the first to propose this “horizontal” form of connection in multi-stream deep learning (DL) architectures. The kind of connection present in U-Nets has been referred to as long skip connections [19] and is an integral part of the models’ ability to prevent dilated features while transferring useful representations to its decoding stage [20]. The other known kind of connection has been referred to as the short-skip connection [19] and was first introduced in ResNet [21] to solve the problem of vanishing gradients as these architectures scaled higher with increasing depths [22,23]. Some research has even combined these connections in their architectural DL designs [24]. The similarity between the long and short connections is the use of simple operations such as addition or concatenation. However, the forms of *feature sharing* range from concatenation to the use of new, joint-processing blocks that can be optimized with the entire architecture. Thus, our research study is novel in its presentation of *feature sharing* between dual-stream architectures. This investigation forms the highlight of our paper, and will be evaluated against the conventional or standalone forms for all the architectures developed.

## 2. Related Work

### 2.1. Behavioural Classification

The seminal work in mouse behavior classification [7] was developed for individually-housed animals, and provides the benchmark dataset with which several other methods (including ours) are evaluated. In this work, a series of hand-crafted shape and motion features were extracted, a support vector machine (SVM) was used alongside a hidden Markov model (HMM) to classify video clips into eight distinct behaviors. Model training was repeated n=12 times using a *leave-one-out* methodology, achieving a classification accuracy of 77.3% across all eight classes as opposed to the 71.6% accuracy by human annotators. However, this work was operated on frame-wise/2D inputs, and does not take-in the entire spatio-temporal context like ours.

Since then, deep learning has emerged as the state-of-the-art for the classification of video data in general. Though 2D (i.e., spatial-only) models thrive in most applications, the need for better contextual understanding has become increasingly apparent, and the application of 3D convolutions has enabled this. Better yet, the use of multiple input streams allows for better encoding of video or clip sequences into different representations. It is often the case that one of the model streams operates on an image or image sequence (within time frame $t_{0}$ to $t_{n}$) while the next stream operates on optical flow data (computed for $t_{1}$ to $t_{n+1}$) [25,26]. Some other multi-stream variations operate on two image streams of different points of view, resolutions [27], or zoom [15] depending on the goal of classification.

The work by [25] presented a new network called the inflated 3D (I3D) Inception model. The I3D modules differed from the classic Inception module [28] due to the addition of 3D convolutions and ‘inflated’ filters that allowed a wider receptive field necessary to better learn spatiotemporal data. This dual-stream I3D architecture (pre-trained on ImageNet) was utilized by [29] to classify home cage mouse behaviors. Its evaluation was carried out on the same dataset [7] and used a *leave-one-out* method, therefore averaging test results across the twelve videos in the main dataset. They achieved an average accuracy of over 90% on testing with various stream weights.

In another paper by [18], the effect of shared features at higher levels of multi-stream networks was demonstrated. The authors termed this operation *feature fusion*. The architecture comprised a frame-wise spatial transformer-based stream and a clip-wise temporal stream. The stream features were combined at two successive final pooling layers, ultimately achieving accuracies of 95.3% on the UCF101 [30] and 72.9% on the HMDB51 [31] datasets. A key difference between this approach and the *feature sharing* approach proposed in this work is its implementation at multiple points throughout the dual-stream architecture (as explained in Section 3.3).

### 2.2. Spatiotemporal Learning

Though utilized in different research, ref. [32] demonstrated that spatiotemporal cuboids of data formed better descriptors in both human activity recognition and mice behavioral classification tasks than spatial-only data. These spatiotemporal features were implied to be better for video classification due to the presence of more information that better captures event contexts, especially those that can be easily confused at instance classification. In rodent phenotyping, a lot of emphasis is placed on themes such as behavioral sequences, periodicity, repetitiveness, or patterns of certain behaviors [33]. Depending on the nature of biological research, these factors become increasingly important to identify, else subtle details are missed. A good example of this is self-grooming behavior, which can be observed as mice transition from their idle periods to high activity [33,34]. However, when in excess, this behavior is also commonly associated with mice models of both autism spectrum disorders and compulsive disorders [35]. This further attests to the importance of capturing temporal content in machine learning models.

One of the key components in deep spatiotemporal learning is I3D [25], as mentioned previously. I3D was built by changing the 2D convolutional layers in the Inception v1 [28] model to 3D convolutions while still leveraging on the efficient structure of the Inception blocks. Unlike other 3D convolutional methods, I3D is deep yet lightweight, and brings the advantages of Inception for static image classification to the spatiotemporal domain. Owing to these advantages, the I3D concept has been applied in some of the architectures proposed in this paper.

#### 2.2.1. Attention Mechanisms

An attention module is characterized by the following elements: query Q, key K, and value V. It attempts to map these to the output and scales the output using the dimension of the keys $d_{k}$. Multi-head attention (MHA) combines multiple attention instances with trainable parameters W and is often utilized to ensure efficient learning of vector sequences [36]. The general expressions for the attention function and multi-head attention are given below:

(1) $$\mathrm{Attention}(Q,K,V)=\mathrm{softmax}(\left({\mathrm{QK}}^{T}\right)/\sqrt{\left(d_{k}\right)})V$$

(2) $$\mathrm{MHA}\left(Q,K,V\right)=\mathrm{Concatenate}\left({\mathrm{head}}_{1},{\mathrm{head}}_{2},\ldots ,{\mathrm{head}}_{i}\right)W^{o}$$

where ${\mathrm{head}}_{i}=\mathrm{Attention}\left({\mathrm{QW}}_{i}^{Q},{\mathrm{KW}}_{i}^{K},{\mathrm{VW}}_{i}^{V}\right)$.

Transformers are a derivative architecture of MHA initially applied to natural language understanding [36] but have also been found to be effective in computer vision. The Vision Transformers (ViT) is one of those that repurposed transformers to image tasks [37]. Further variations of ViTs designed for spatiotemporal learning of videos have achieved state-of-the-art (SOTA) results in activity recognition [38,39]. The work by [38] also proved that multi-head attention captures vital temporal dependencies by focusing on displaced or moving objects within a sequence. Furthermore, its application was proven to be effective in capturing global features in a multi-stream architecture for video classification [40].

#### 2.2.2. Long-Short Term Memory (LSTM)

LSTMs are architectures that learn to store information using memory cells and gates. The memory cell was designed to achieve constant error flow and used multiplicative input and output gates that protect data from perturbation [41]. Further improvements after this saw better-defined gate operations which improved the memory retention of the architecture.

Building upon this, Bidirectional LSTMs (BiLSTM) allow for the computation of memory both ways and have been proven to achieve good results in vision tasks. A BiLSTM is composed of two LSTMs that store relevant dependencies from both forward (i.e., past to present) and backward (i.e., future to present) state directions [42]. In conjunction with other ML architectures, bidirectional LSTMs have been found to outperform the unidirectional LSTM in several natural language understanding [43,44] and image classification [45] tasks. In the paper by [42], a BiLSTM was used with 1-dimensional convolutions to classify the circadian rhythm of wild-type mice into day or night states. This was trained after the dimensionality reduction of a five-minute clip which was further subdivided into three-second frame windows. It was found to outperform the other ML algorithms explored, achieving an area-under-the-curve (AUC) of 0.97. In short, BiLSTMs are capable of efficiently detecting and learning patterns that define the behaviors mapped.

## 3. Materials and Methods

### 3.1. Data

The MIT mice dataset [7] is subdivided into a main dataset and clipped database. In this work, we utilize all twelve videos from the main dataset for training and validation while the clipped database, composed of unambiguous behaviors, is used to test the models. Specifically, the video recordings from the *20080423191834F* folder were used for validation, while the rest of the main data were used for training. Unlike the *leave-one-out* methodology by the original authors, we surmise that this approach helps us to better examine the generalization performance of our models. Nonetheless, we also made comparisons to the original cross-validation results. The optical flow data were generated from the raw videos using the dense optical flow method [46]. Both training and test frames were resized to 128×128, and further reduced to 128×96 by uniformly cropping redundant parts of each frame that lie along the vertical axis. The data were also temporally downsampled using five-frame intervals. The temporal length used for each *T* = 8 frames. Thus, each spatiotemporal cuboid represents approximately a 1.33-second window in the original videos. Toward the end of the videos/clips, any frames that could not fit these specifications were discarded. The final input data are in the form N×T×W×H×C which represents the number of clips, temporal length, spatial width, spatial height and number of channels respectively. The *N* values for the final training, validation, and testing sets are 23,444, 4195, and 5171 respectively.

### 3.2. Pre-Processing

**Figure 2:**
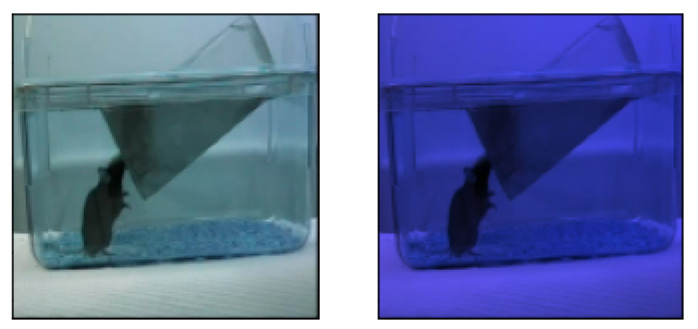
Sample frame before and after ‘*nightification*’.

Class imbalance was alleviated using class weights [47], which forced the model to perceive the number of samples in each class as having the same value. Hence, the classes that suffered from low sample sizes, such as *drinking*, were assigned higher weights, and the reverse for labels with large sample sizes like *micromovement*. Additionally, pre-processing was carried out to normalise visual differences present between videos acquired at different times of day. In this particular dataset, there are only two videos recorded at night-time (using infrared cameras) while all the rest were recorded during the day. In some deep learning applications, conversion to grayscale has proven effective but this method was found to degrade the performance of the models. As such, all day videos contained within the dataset were ‘*nightified*’ (i.e., changed into night-time). This was achieved by first calculating the averaged R, G, and B channel values from the night videos. These were then used to weigh the [0–1] normalized data from the day videos and finally expanded back to the [0–255] range. The results gave a close approximation of what the videos would look like if recorded at night, and thus lessened bias in the models caused by the day-night imbalance (Figure 2). No further augmentations were performed on the dataset. More data samples, used in both RGB and flow streams, are available in Appendix D.

### 3.3. Architectures

**Figure 3:**
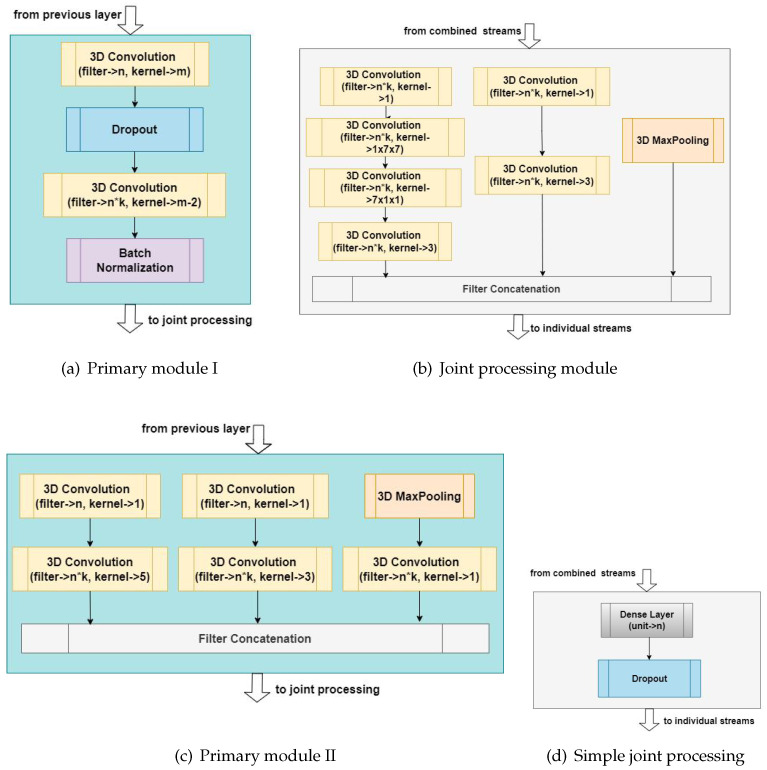
Primary and *feature sharing* modules used in the architectures. Note that the variables *k*, *n*, and *m* used here represent the multipliers, filter sizes, and kernel sizes respectively, at different levels in the architectures.

All of the models presented are dual-stream and, in this application, use raw video and optical flow streams. The building blocks utilized in the networks are depicted in Figure 3. One of the more vital aspects of the models presented here is the *feature sharing* between the dual-streams of the network. *Feature sharing* involves the combination and/or joint processing of the stream outputs after operation by the primary modules. This combination is achieved either via addition or concatenation, followed by further processing on the joint streams which are then projected back to the individual streams. These operations take place at regular intervals throughout the architecture. We hypothesize that this procedure reinforces learned features better than operating on the streams individually. The various implementations of these modules are further discussed under each architecture and in Table 1. The overview of each architecture is also depicted in Appendix E.

The blocks in Figure 3a,c represent the primary processing modules used in both the RGB image and optical flow streams, while the blocks in Figure 3b,d are the joint processing modules. The blocks in Figure 3b,c depict the 3D formats of modules originally found in Inception v3 and Inception v1 architectures respectively [28,48]. In particular, Figure 3b was adapted here to boost the performance of the architectures utilizing module Figure 3a via further processing at the junctions where the streams meet. Block Figure 3d is a custom joint processing module utilized only in the baseline network.

#### 3.3.1. Baseline Network (CNN)

This simple architecture consists of blocks with 3D convolutional layers, dropout (with uniform rates of 20%), and batch normalization (see Figure 3a). The kernel sizes here were made uniform for each block (i.e., kernels of size *m* rather than m−2 as depicted in Figure 3a). After operation by similar blocks, the results from both streams are summed up and further operated on by dense and dropout layers (Figure 3d) before splitting again into the individual streams.

#### 3.3.2. CNN + Inception v3_D + Attention (CIv3D_MHA)

This builds on the baseline architecture, adding the self-attention mechanisms to both streams after the last primary blocks. The kernel size for 3D convolutions was made to increase and decrease repeatedly (as shown in Figure 3a) between stream blocks. In addition, the simple processing block is replaced by the Inception v3 block D [48] (Figure 3b) throughout the architecture. The self-attention block used here is similar to vision transformers [37] however it uses batch normalization, and the patch tokens are replaced by the end features of the streams before summation and processing by the last InceptionD block.

#### 3.3.3. CNN + Inception v3_D + BiLSTM (CIv3D_BiLSTM)

This uses the same improvisations made in CIv3D_MHA but removes the primary modules’ dropout layers. The bidirectional LSTMs are used in place of the traditional flattening that precedes fully-connected (FC) layers. The input to this is the summed output of both streams’ final subsection, reshaped from four to two dimensions to allow loading into the LSTMs.

#### 3.3.4. Purely Inception-Based Networks

There are two architectures completely built up using the 3D Inception v1 block (see Figure 3c). This block was revised for spatiotemporal operation from the dimensionality reduction module in the classic Inception v1 architecture [28] but is without the singular 1×1 convolution branch in the original (Figure 3c). The first architecture works by reinforcing representation learning in a single stream rather than splitting the features apart. At the bottleneck between successive sub-regions of the network, feature learning is reinforced by repeatedly concatenating strided computations of the original optical flow sequence with the previous features extracted from the RGB stream. Hence, the network was termed the Singly Reinforced Stream (SRS) network. It also adds the design consideration of removing the first and last two frames of the optical flow stream (along with some surrounding dimensions) after the first block operation on both streams. This cropping operation is carried out only once and under the assumption that the temporal sequence is better represented by the center portions of the mid-four frames. This train of thought is quite similar to the fovea stream in [15] but takes it further by removing frames at the extremities.

The second architecture was developed to encourage cross-pollination between streams; this implies that just as the optical stream enforces representation learning in the image stream, the image stream is also used to enforce learning in the optical stream, and they alternate in this manner. This is carried out by independently concatenating the past features from each streams’ block with the jointly-processed input fed into consequent blocks. This operation however led to a considerable increase in computation (see parameter count in Table 2). This network was named Cross Reinforced Streams (CRS) network.

#### 3.3.5. Other Networks

To investigate the effectiveness of the shared layers between streams, experiments were conducted on versions of the above models without the unique *feature sharing* modules. The design considerations used in each architecture were left in place while the blocks of joint processing (i.e., *feature sharing*) were replicated in both streams, all before the common fully-connected layers.

### 3.4. Model Training

All models were trained using the categorical cross-entropy loss and optimized using stochastic gradient descent (SGD). The number of epochs and batch size were set to 85 and 8, respectively. Training was set to reduce its learning rate by a factor of 0.5 if validation loss plateaus or peaks, and to finally stop if no notable learning is achieved. This prevents overfitting and allows for the early restoration of the best checkpoints. Each model is trained and evaluated n=4 times corresponding to different random seeds, and averaged. By using the averages, we present an accurate representation of each models’ predictive capability. The system used for all the experiments was equipped with 64GB RAM and 2 Nvidia GeForce RTX-3080 GPUs.

### 3.5. Metrics

The most popular metric used for classification problems is accuracy. However, we evaluate all the models presented here on several metrics, including accuracy, average precision (AP), precision, recall, F1 score, and area-under-the-ROC-curve (AUC), where ROC is the receiver operating characteristic. More specifically, the AP metric (which was utilized the most) is the micro-averaged precision, while the precision is the macro-averaged per-class computation. All together, these metrics give a holistic view of each models’ performance.

## 4. Results

### 4.1. Model Comparison

The results of all seed models for each of the architectures were averaged to achieve the final results. The full performances for each seed can be found in Appendix A. The best result was obtained for the singly-reinforced stream (SRS) architecture, achieving an average accuracy of 81.96 ± 2.71%. The averaged performances of all the *feature sharing* dual-stream models are tabulated in Table 3.

### 4.2. Ensembles

The ensembles were created by averaging the results of the models at inference. Due to the gap in performance, most ensembles between models did not show any improvements over the SRS model. The final choice of models to ensemble was made by evaluating the validation results for all seed training in each model. For intra-model ensembles (that is, between the top 2 seeds of the same model), the best results were found for the SRS model and achieved 82.37%. The best inter-model ensemble was observed between the SRS and CIv3D_MHA models and achieved 86.28%. Further ensembles between models are shown in Table 4. The confusion matrices and ROC plots for the ensembles can be found in Appendix B.

### 4.3. Ablation Study

#### 4.3.1. The Case for *Feature Sharing*

Here, the results of the models and their non-*feature sharing* variants are presented. The variants were trained and tested on the same dataset, and under the same conditions as those with joint processing. The averaged results across all metrics are tabulated (Table 5). In general, only the accuracy shows significant variation between seed models while the variation in other metrics is negligible. It can be clearly observed that for each architectural pair (i.e., *feature sharing* vs standalone), the *feature sharing* models perform better than their standalone forms.

The performance gains are especially pronounced in the Inception-based architectures with the SRS gains ranging from 6.59% to 15.19%, and the CRS gains ranging from 7.79% to 13.29%. The lowest observable gain after applying *feature sharing* was a 0.33% increase in accuracy, calculated for the baseline model. Conversely, the only occasion of a loss in accuracy (after *feature sharing*) was observed for the CIv3D_BiLSTM model having a value of −3.66%. Nonetheless, this same architecture was also found to be able to achieve gains of about 3.92% over its standalone streams.

#### 4.3.2. The Case for *nightification*

To justify the choice of *nightified* spatiotemporal (ST) clips in the image stream, further experiments were conducted for both raw RGB input and grayscale input. These experiments were carried out on the baseline model and the previously ascertained best-performing models from Section 4.2. These models were trained and tested in the same rigorous manner as the core paper models. The results show that *nightified* ST input has higher accuracy than both grayscale and raw video ST inputs for most models, the only exception being the baseline model. Those using grayscale cuboids seemed to initially perform well just observing the AUCs and average precision however all their accuracies were subpar compared to the *nightified* cuboids. Observations show that this was due to greater misclassification between visually similar behaviors (such as *micromovement* and *rest*), indicative of the fact that the grayscale modality did not possess sufficient information for these deep models to distinguish between the behaviors. A similar narrative was observed in the raw video inputs though we argue that, in this case, the drop in performance (albeit small) was due to the lack of standardization. The results are presented in Table 6.

#### 4.3.3. Varying Temporal Length

The temporal length refers to the number of frames that make up each clip. As previously stated, all architectures were designed for a temporal length *T* = 8, corresponding to 1.33 seconds. Further experiments are performed here by varying the preset *T* value. The new temporal lengths chosen were (i.e., *T* = 4) and (i.e., *T* = 16). These experiments were only carried out on the baseline and SRS models, and were conducted in the same rigorous manner as the initial runs. Besides changing the input shape, the temporal cropping (refer to Section 3.3.4) in the SRS architecture was also slightly modified. Same as the new *T* values, this feature was halved and doubled respectively for *T* = 4 and *T* = 16. Hence, there was no change to the architectural complexity. For the baseline model, its complexity only increased, slightly, for *T* = 16. The results after averaging the results for various seeds are shown in Table 7.

The results show that the preset *T* = 8 was optimum as the accuracies obtained in the new experiments were not up to par. In order of performance, the models having input temporal dimensions of *T* = 8 were the best, followed by *T* = 4 and lastly *T* = 16.

#### 4.3.4. Cross Validation

As stated in the review section, the authors of the original dataset performed cross-validation using the main mice dataset comprised of the twelve videos in the main dataset. Here, the same n=12 cross-validation is carried out and the results are reported for the best ensemble in Section 4.2, comprised of the SRS and CIv3D_MHA models. The final results are shown in Table 8.

The result achieved is seen to perform better than the human annotators; however, it is lower than the proposed method in the original paper. Despite the difference in model contexts (i.e., spatiotemporal against their framewise model), the results achieved ascertain the validity of our methodology. However, unlike the original publication [7], all our models are trained from scratch and have no prior contact with the MIT main dataset.

### 4.4. Other Datasets

#### SCORHE

Further experiments were conducted by applying the pre-trained versions of the top three seeds (from all models) to a different home-cage mouse dataset [10]. As previously shown in Section 4.2, the top performing seeds (as identified by the validation data) occur in these models: CIv3D_BiLSTM, CIv3D_MHA, and SRS. Although 13 unique annotations were originally present (see graph in Appendix C), these were refined to 8 classes by removing samples with ambiguous classes (such as *behav_ignore, behav_other*), removing samples having extremely low class occurrence (such as *discrepancy, rotating*), and merging the supported and unsupported *rearing* classes.

The recordings in the SCORHE home cage were captured from multiple points as no singular viewpoint provides a clear view due to occlusions. To address this, the viewpoints from opposite ends of SCORHE were shaped as 128 × 64 frames and stacked into a singular 128×128 frame. The same was also performed for the optical flow data stream. No frame skips were used here to ensure ample training and testing data were available. Data samples for the SCORHE dataset are available in Appendix D.

**Figure 4:**
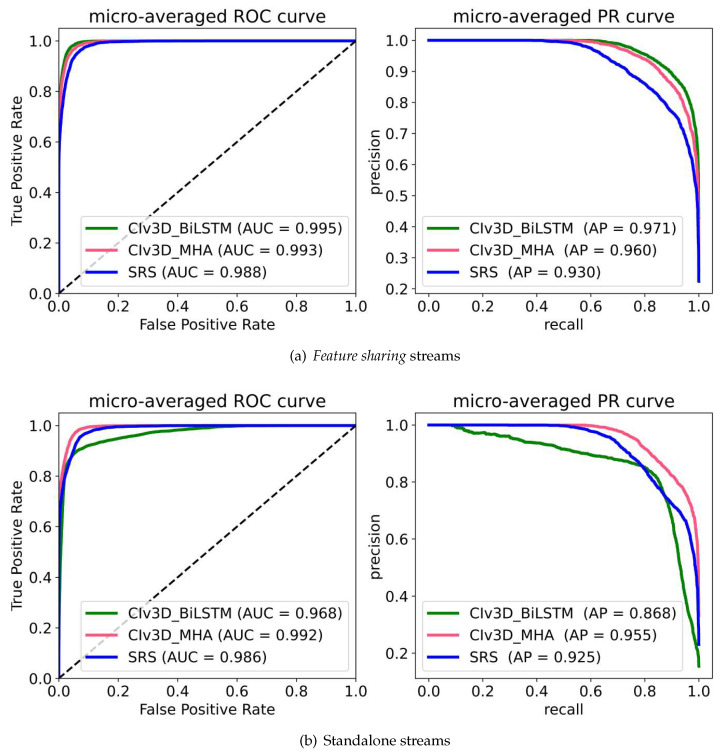
ROC and PR curves between the *feature sharing* and conventional architectures using the SCORHE dataset. On observation, all the *feature sharing* architectural forms were found to also outperform their standalone stream variants in both AUC and average precision.

For the training, the previous FC layers were replaced and all other training parameters were kept the same, except for the learning rate which was halved to 0.0005. The resulting receiver operating characteristics (ROC) and precision-recall (PR) curves are shown in Figure 4. The accuracies achieved on the SCORHE dataset by the *feature sharing* CIv3D_BiLSTM CIv3D_MHA and SRS were 80.51%, 79.88% and 79.13% respectively. Their non-*feature sharing* variants achieved 72.18%, 77.95% and 70.83% respectively.

A few observations were made on the *feature sharing* models. The CIv3D_BiLSTM and CIv3D_MHA were good at reinforcing previously learned spatiotemporal representations to this complex home cage for similar behaviors. However, despite having lower accuracy, SRS performed better in both learning old classes and balancing predictions to learn totally new class, *climbing*. This is proven by its class accuracy across the different confusion matrices; while CIv3D_BiLSTM and CIv3D_MHA achieved 22.34% and 33.68% respectively, the SRS model achieved 53.61%.

## 5. Discussion

Generally, it was observed that the more dynamic behaviors were better captured, by all the models, than the less dynamic behaviors. Areas of weak performance across all the models were mainly due to misclassification of *resting*, *grooming*, and *micromovement* behaviors. These behaviors are quite closely related; during *grooming*, the mouse is mostly stationary albeit the motion of its forelimbs and when resting, the mouse is completely immobile. *Micromovement* describes very small-scale motions and hence it is most likely that the 1.33-second windows of *T* = 8 cuboids cannot capture the full range of motion to distinguish between these classes. Nonetheless, these ’misclassifications’ are also indicative of similitude in the temporal pattern needed to perform certain tasks and may be subject to further interpretation by the subject experts.

Further experiments in the ablation study also showed that, for time windows lower or higher than the 1.33-second window, the performance of the models degrades. Thus, other clip sizes will require more intense hyperparameter tuning and data preprocessing to work with the *feature sharing* paradigm. In particular, the *T* = 16 temporal input may also require a deeper architecture (i.e., having more rungs or blocks) at the cost of increasing the computational complexity of the learning objective. The step up in performance between the *feature sharing* and standalone baseline models lends credence to the effectiveness of combined streams; by simply summing parallel outputs from both streams and processing with a dense-dropout pair (depicted in Figure 3b), we observe between 0.33% and 8.97% improvement in accuracy. This observation was further proven in subsequent networks utilizing algorithms such as bidirectional LSTMs and self-attention mechanisms. Though the CIv3D_BiLSTM model was only marginally better in terms of accuracy, it outperformed its non-*feature sharing* variant in all other metrics. Similarly, we observe a notable improvement across all the metrics for the other models, especially in the purely 3D Inception-based networks (SRS and CRS), both having over 10% improvement in averaged accuracy alone. The ensemble of the SRS and CIv3D_MHA was also seen to achieve better accuracy than human annotators on cross-validation using the training data. Although this accuracy was not up to par with the proposed methodology in the original paper, it sufficiently demonstrates the workability of the *feature sharing* paradigm.

Based on both the parameter count and floating point operations per second (FLOPS), the implementation of *feature sharing* was also found to mostly reduce the complexity of the architectures, with the exception of the CRS model (see Table 2). Conversely, utilizing *feature sharing* would require establishing which *feature sharing* method is best suited for the architecture, i.e., either simple concatenation or a new processing block (such as Figure 3d or Figure 3b). These investigations would generally increase the number of experiments needed, therefore increasing the time needed to establish its utility.

## 6. Conclusions

In summary, this paper proposed an approach to mouse behavior classification based on multi-stream convolutional neural networks with *feature sharing*. By including this architectural consideration, we observed gains ranging from 0.33% to 15.19% for all the custom architectures that were presented. Only in one model type (i.e., the CIV3D_BiLSTM) was the *feature sharing* architecture reported to achieve a lower accuracy than its standalone variant. Nevertheless, upper-limit gains of 3.92% were also possible for this same architecture. We validate this approach using two publicly available datasets, and it performs favourably compared to the start-of-the-art.

Further work will investigate improving the overall cross-validation by employing data augmentations not employed in this paper. In addition, *feature sharing* can be adapted using well-established, state-of-the-art supervised models (both convolutional and transformer-based) to further investigate its pros and cons. Finally, future research will also consider the unsupervised detection of behaviors and welfare concerns in the home cage, and whether the unique *feature sharing* approach will impact multi-stream models in this learning domain.

## Figures and Tables

**Table 1:** Full summary of *feature sharing* models showing the internal parameters and output sizes for each stacked module.

**Models**	Baseline	CIv3D_BiLSTM	CIv3D_MHA	SRS	CRS
**Primary filters (n) ${}^{1}$**	16, 32, 64, 128	16, 32, 64, 128	16, 32, 64, 128	8, 16, 32, 64, 64,128	12, 24, 48, 96, 192
**Intra-block filter multipliers (k) ${}^{1}$**	1.5	1.5	1.5	1.5, 1.5, 1.5, 1.5, 1.5, 1.0	1.0
**Kernels (m)**	5, 3, 5, 5	7, 5, 5, 3	7, 5, 5, 3	refer to Figure 3c	refer to Figure 3c
**Stream combination via?**	Addition	Addition	Addition	Concatenation	Concatenation
**Processing at joints?**	Yes (refer to Figure 3d)	Yes (refer to Figure 3b)	Yes (refer to Figure 3b)	No	No
**Further processing before FC?**	No	No	No	Single Inception v1 block (Figure 3c)	No
**Activation function(s) before FC?**	Leaky ReLU	Leaky ReLU	Leaky ReLU	ReLU	ReLU
**Activation at last dense layer?**	Softmax	Softmax	Softmax	Softmax	Softmax
**FC units (Descending)**	512, 64, 8	512, 64, 8	512, 64, 8	512, 64, 8	512, 64, 8
**Output sizes after joint processing blocks ${}^{2}$**					
**Module 1**	4 × 24 × 32 × 24	4 × 24 × 32 × 24	4 × 24 × 32 × 24	8 × 96 × 128 × 72	8 × 96 × 128 × 72
**Module 2**	2 × 12 × 16 × 48	2 × 12 × 16 × 48	2 × 12 × 16 × 48	4 × 48 × 64 × 144	4 × 48 × 64 × 144
**Module 3**	1 × 6 × 8 × 96	1 × 6 × 8 × 96	1 × 6 × 8 × 96	2 × 24 × 32 × 288	2 × 24 × 32 × 288
**Module 4**	1 × 3 × 4 × 192	1 × 3 × 4 × 192	1 × 3 × 4 × 192	1 × 12 × 16 × 576	1 × 12 × 16 × 576
**Module 5**	-	-	-	1 × 6 × 8 × 576	1 × 6 × 8 × 1152
**Module 6**	-	-	-	1 × 3 × 4 × 384	-

${}^{1}$ filter/unit size n in joint processors (Figure 3b,d) is based on filter size after stream combination (i.e., adding or concatenation) and uses fixed multiplier k = 1.5. ${}^{2}$ Striding through temporal dimension produced more compact feature representations and lessened overall parameter count.

**Table 2:** Learning rates, parameter count, and floating point operations per second (FLOPS) for each model.

**Models**	Baseline	CIv3D_BiLSTM	CIv3D_MHA	SRS	CRS
**Learning Rate(s)**	0.0005	0.001	0.001	0.001	0.001
**Parameters (** ***feature sharing*** **)**	11,315,848	13,243,712	15,554,112	9,671,872	22,927,824
**Parameters (standalone)**	11,348,728	16,623,752	19,044,744	9,670,024	22,483,944
**FLOPS (** ***feature sharing*** **)**	$22.63\times 10^{6}$	$30.67\times 10^{6}$	$31.10\times 10^{6}$	$19.34\times 10^{6}$	$45.85\times 10^{6}$
**FLOPS (standalone)**	$22.69\times 10^{6}$	$37.42\times 10^{6}$	$38.07\times 10^{6}$	$19.34\times 10^{6}$	$44.96\times 10^{6}$

**Table 3:** Performance of proposed models across chosen metrics showing that the SRS outperforms all other architectures across all metrics.

Models	Metrics					
	AUC		Precision	Recall	F1-Score	Accuracy
	micro (m)	macro (M)				
Baseline	0.879	0.920	0.608	0.749	0.607	74.93
CIv3D_BiLSTM	0.955	0.965	0.731	0.771	0.687	77.08
CIv3D_MHA	0.951	0.953	0.652	0.780	0.671	77.99
SRS	0.959	0.972	0.796	0.820	0.750	**81.96**
CRS	0.934	0.960	0.755	0.785	0.683	78.49

**Table 4:** Result of binary ensembles between SRS, CRS, CIv3D_MHA and CIv3D_BiLSTM models.

Ensembles	Metrics		
	mAUC	AP	Acc (%)
SRS + CRS	0.958	0.795	83.31
SRS + CIv3D_MHA	0.977	0.880	**86.28**
SRS + CIv3D_BiLSTM	0.966	0.830	83.69
CRS + CIv3D_MHA	0.963	0.814	82.62
CRS + CIv3D_BiLSTM	0.942	0.745	79.55
CIv3D_MHA + CIv3D_BiLSTM	0.968	0.831	82.46

**Table 5:** Detailed comparison between the *feature sharing* and standalone stream forms of each network. The standard deviation (from the mean metric value) across the four different seed models in each case was also included.

Models	Stream Kind	mAUC	MAUC	AP	F1 Score	Accuracy (%)
Baseline	sharing	0.878 ± 0.013	0.920 ± 0.004	0.592 ± 0.017	0.552 ± 0.026	74.93 ± 2.68
	standalone	0.815 ± 0.020	0.916 ± 0.010	0.562 ± 0.024	0.483 ± 0.016	70.28 ± 1.64
CIv3D_BiLSTM	sharing	0.955 ± 0.012	0.965 ± 0.003	0.789 ± 0.045	0.654 ± 0.074	77.08 ± 1.77
	standalone	0.910 ± 0.015	0.955 ± 0.004	0.668 ± 0.051	0.537 ± 0.026	76.95 ± 2.02
CIv3D_MHA	sharing	0.951 ± 0.017	0.953 ± 0.017	0.770 ± 0.060	0.678 ± 0.074	77.99 ± 3.50
	standalone	0.896 ± 0.018	0.938 ± 0.007	0.635 ± 0.036	0.564 ± 0.034	73.10 ± 2.44
SRS	sharing	0.959 ± 0.010	0.972 ± 0.006	0.791 ± 0.042	0.686 ± 0.050	81.96 ± 2.71
	standalone	0.900 ± 0.029	0.931 ± 0.009	0.632 ± 0.064	0.666 ± 0.048	71.07 ± 1.59
CRS	sharing	0.934 ± 0.006	0.960 ± 0.004	0.728 ± 0.015	0.578 ± 0.018	78.49 ± 0.95
	standalone	0.868 ± 0.016	0.921 ± 0.008	0.562 ± 0.025	0.523 ± 0.019	67.95 ± 1.80

**Table 6:** Results on grayscale (GS), raw RGB (R), and *nightified* (N) data show that 3-channel inputs in the raw image stream produced a better performance than the single channel grayscale in all the architectures considered. Of these models, two of the best performers were associated with the *nightified* data format.

Model	Baseline			CIv3D_MHA			SRS		
**Input kind**	GS	R	*N*	GS	R	*N*	GS	R	*N*
**mAUC**	0.873	0.869	0.879	0.910	0.921	0.950	0.962	0.938	0.959
**MAUC**	0.910	0.914	0.920	0.929	0.943	0.953	0.972	0.953	0.972
**AP**	0.545	0.577	0.592	0.647	0.673	0.770	0.810	0.687	0.791
**F1 Score**	0.542	0.544	0.552	0.564	0.597	0.678	0.733	0.569	0.686
**Accuracy (%)**	73.37	**76.57**	74.93	73.70	77.16	**77.99**	9.24	77.87	**81.96**

**Table 7:** Results for varied temporal lengths show that the 1.33-second window was optimal for the depth and complexity of the architectures presented.

	Temporal Length (s)	mAUC	MAUC	AP	F1 Score	Accuracy (%)
**Baseline**	4	0.853	0.743	0.411	0.381	57.03
	8	0.879	0.920	0.592	0.552	**74.93**
	16	0.831	0.736	0.365	0.349	47.63
**SRS**	4	0.914	0.958	0.629	0.576	69.72
	8	0.959	0.972	0.791	0.686	**81.96**
	16	0.916	0.930	0.658	0.607	67.43

**Table 8:** Result on cross-validation only shows results comparable to those of human annotators.

	Cross-Validation Acc (%)
Human Annotators ([7])	71.6
Their method ([7])	77.3
Ours	71.8

**Figure A1:**
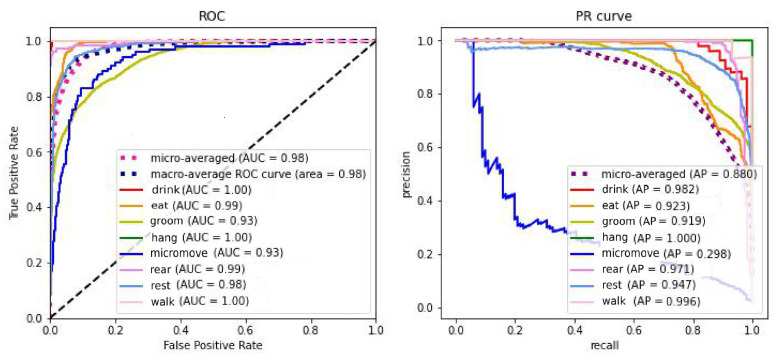
ROC and PR plot for SRS + CIv3D_MHA.

**Figure A2:**
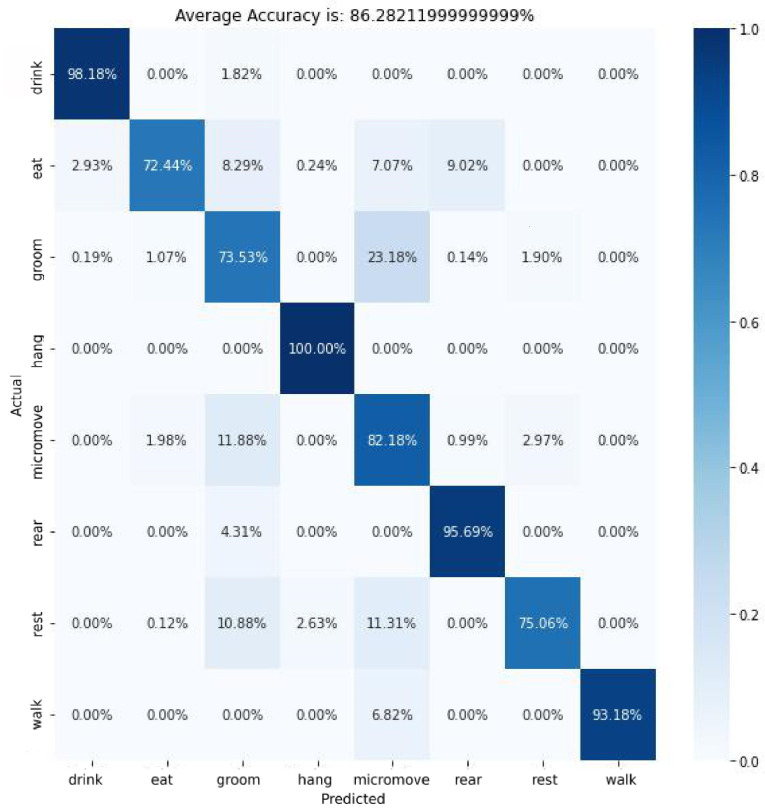
Confusion Matrix for SRS + CIv3D_MHA.

**Figure A3:**
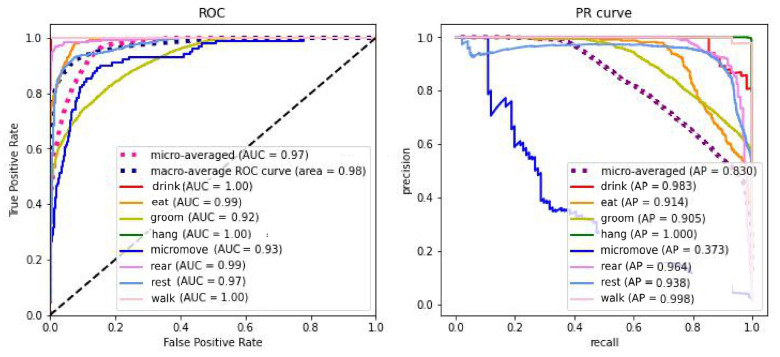
ROC and PR plot for SRS + CIv3D_BiLSTM.

**Figure A4:**
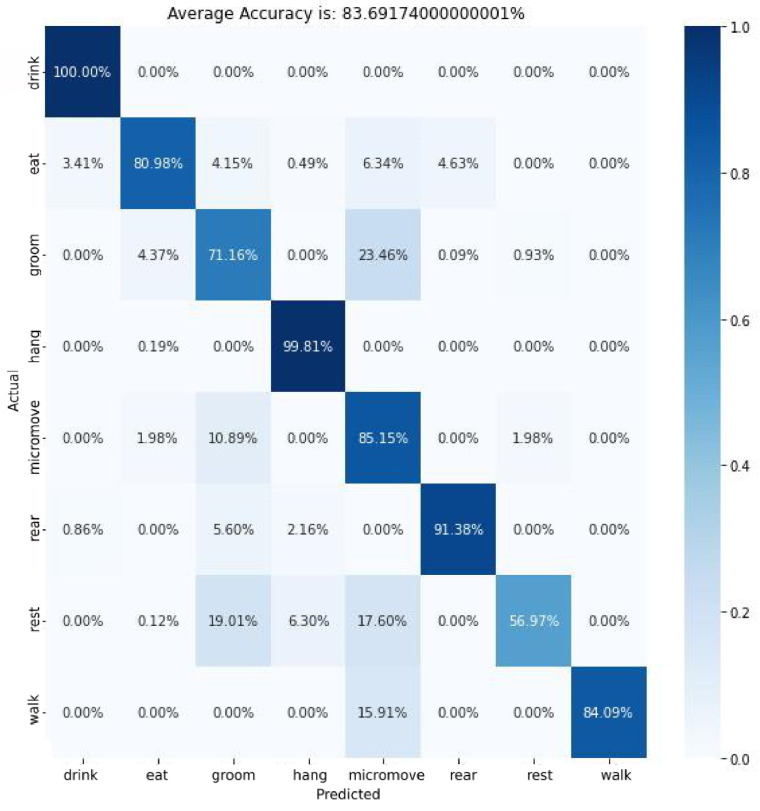
Confusion Matrix for SRS + CIv3D_BiLSTM.

**Figure A5:**
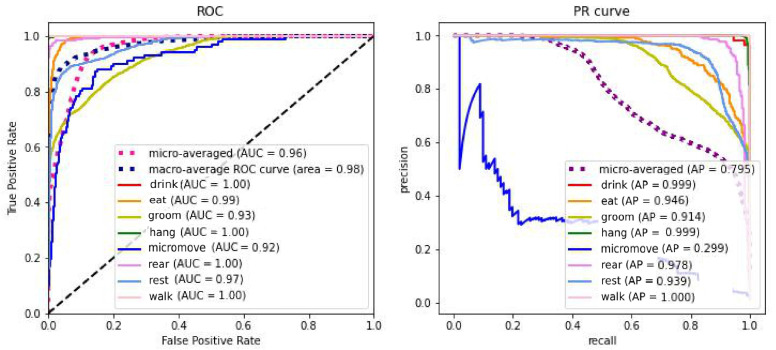
ROC and PR plot for SRS + CRS.

**Figure A6:**
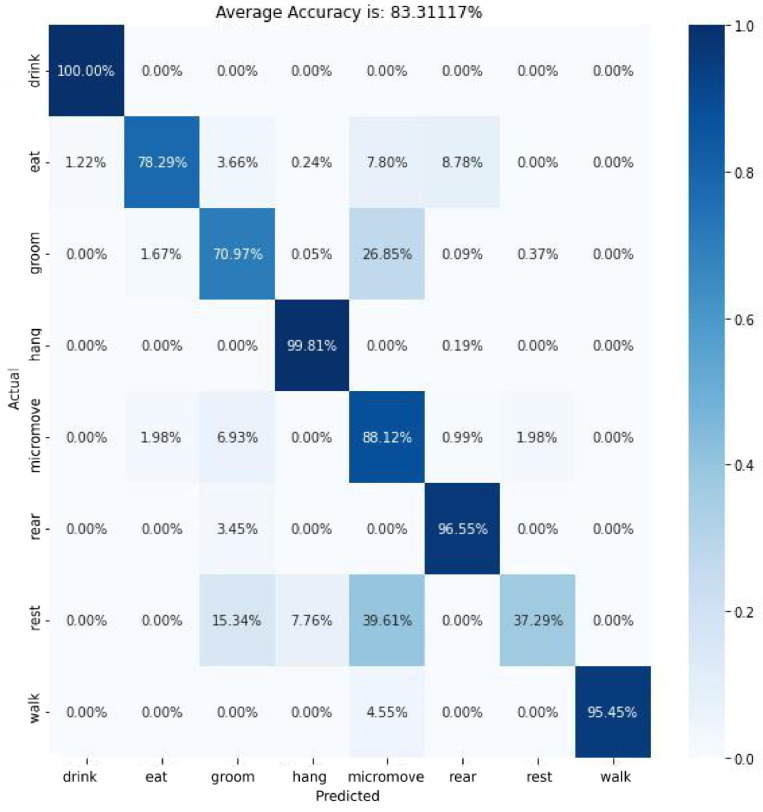
Confusion Matrix for SRS + CRS.

**Figure A7:**
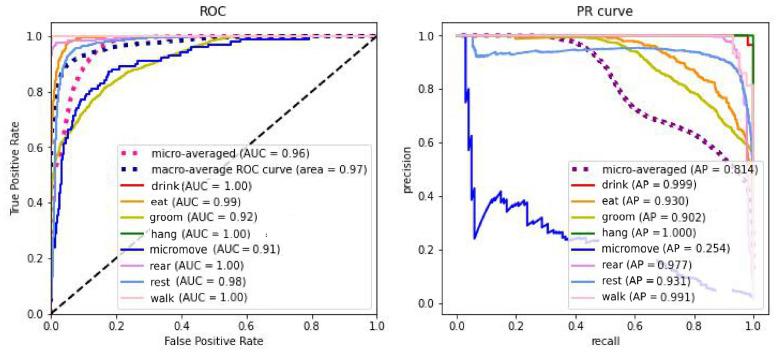
ROC and PR plot for CIv3D_BiLSTM + CIv3D_MHA.

**Figure A8:**
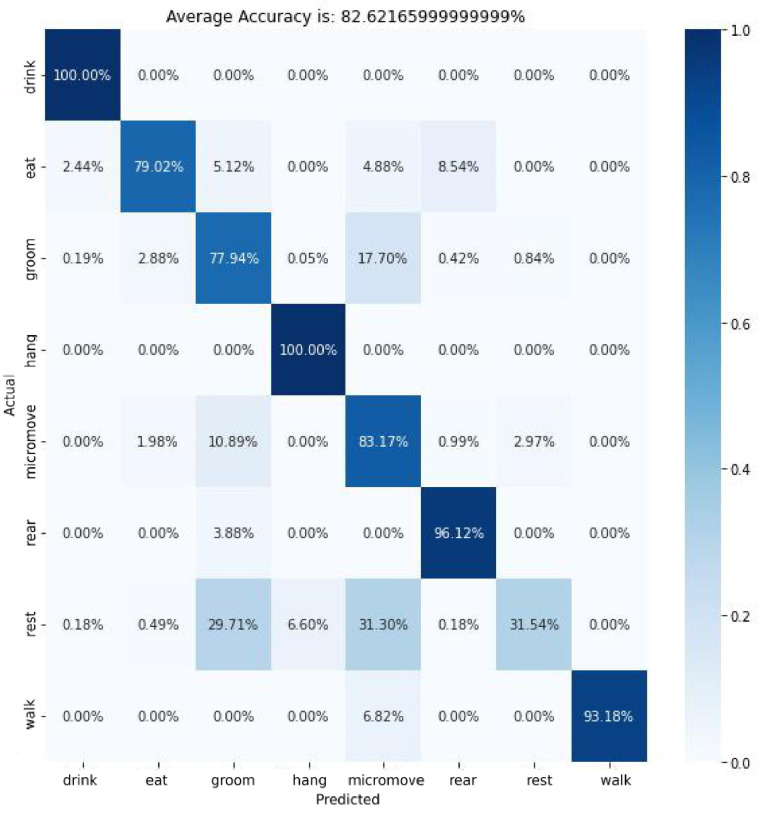
Confusion Matrix for CIv3D_BiLSTM + CIv3D_MHA.

**Figure A9:**
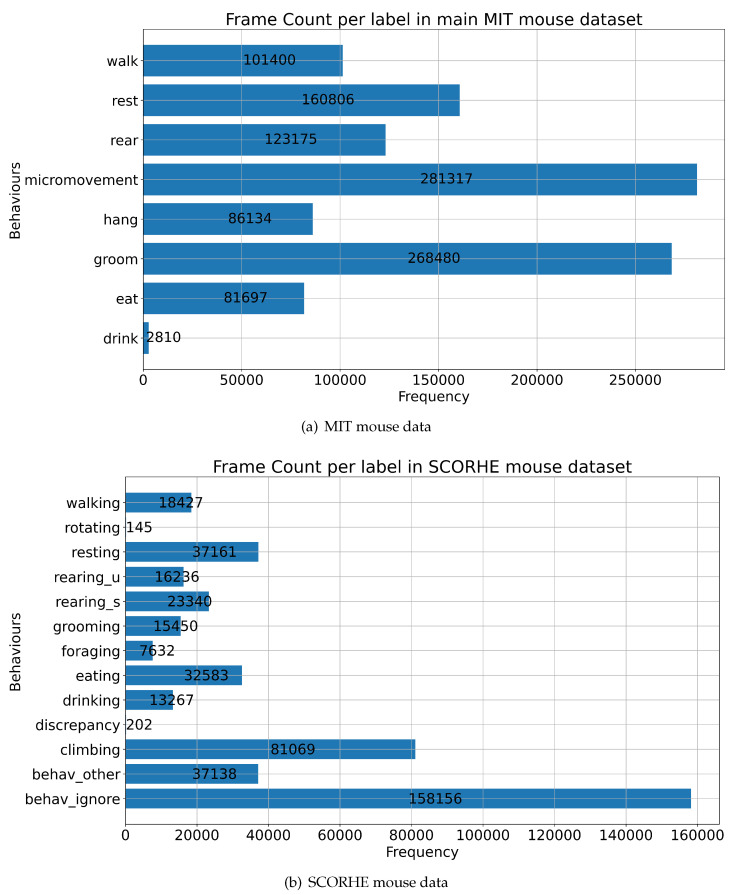
Full data summaries (before preprocessing).

**Figure A10:**
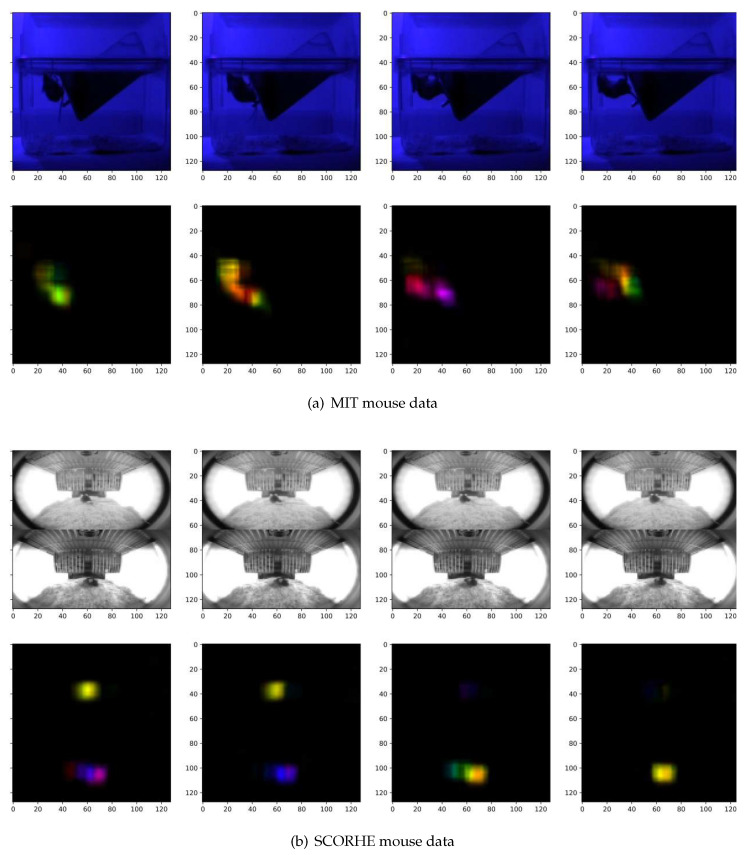
Samples from SCORHE and MIT dataset.

**Figure A11:**
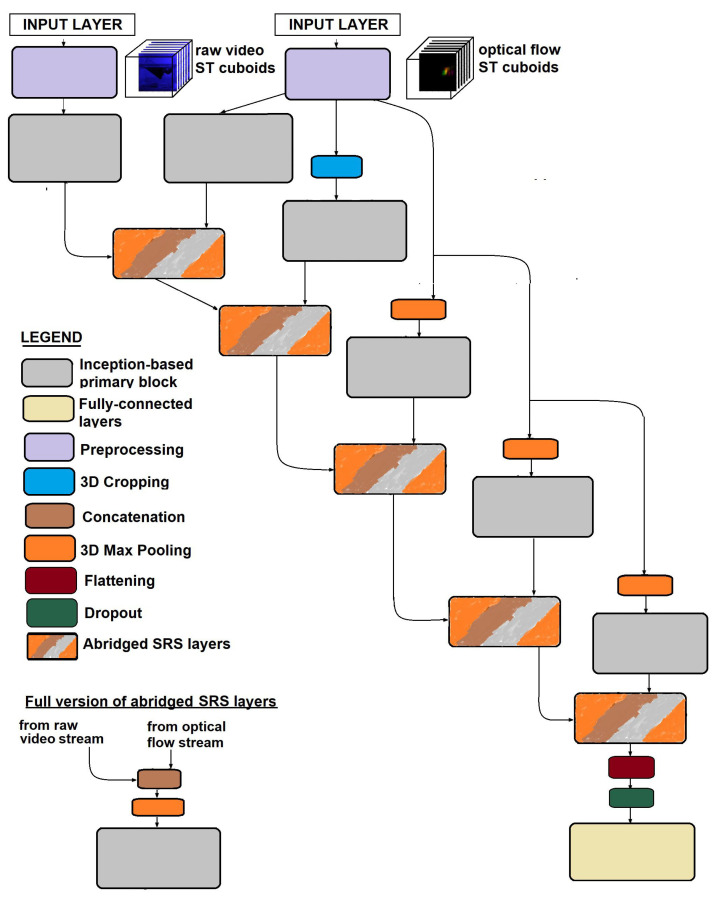
Overview of SRS model.

**Figure A12:**
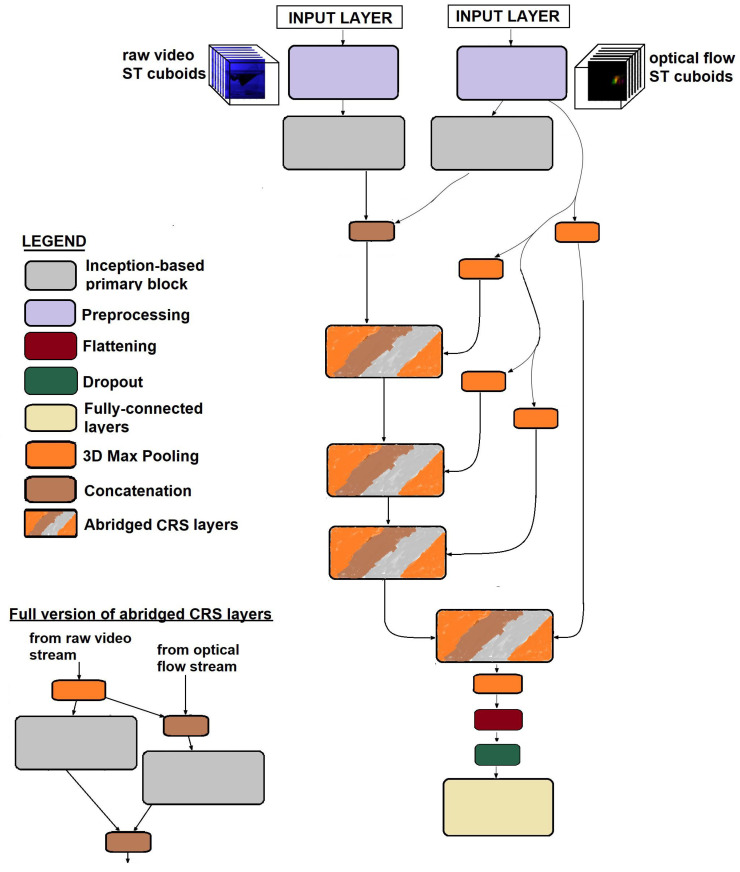
Overview of CRS model.

**Figure A13:**
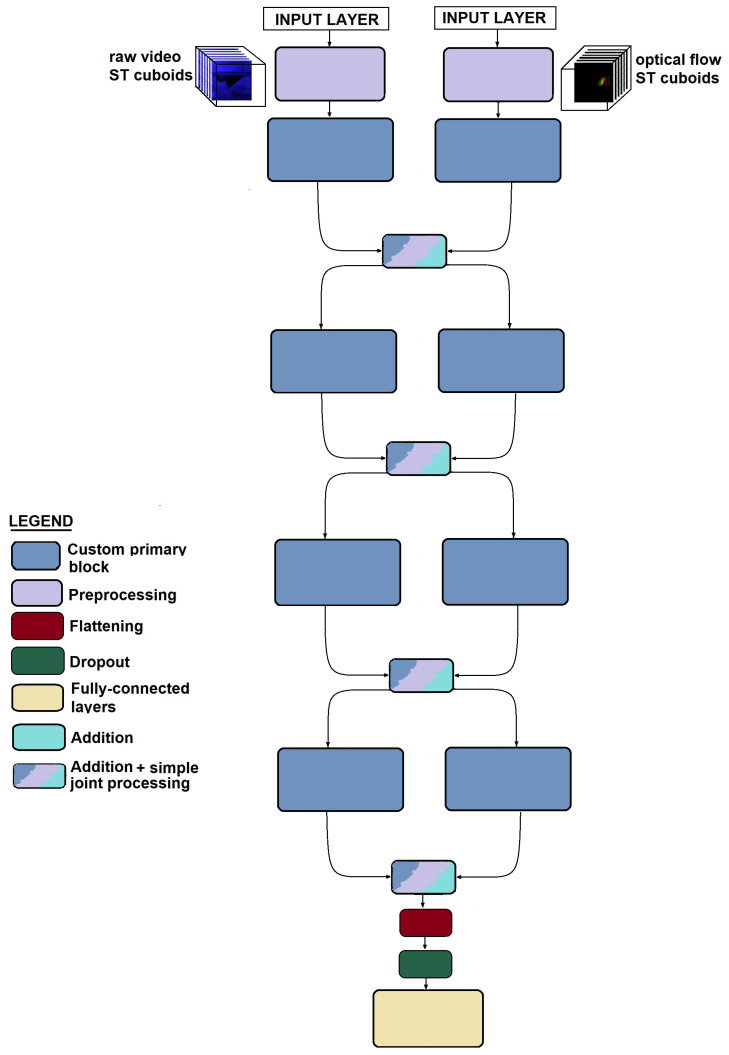
Overview of Baseline model.

**Figure A14:**
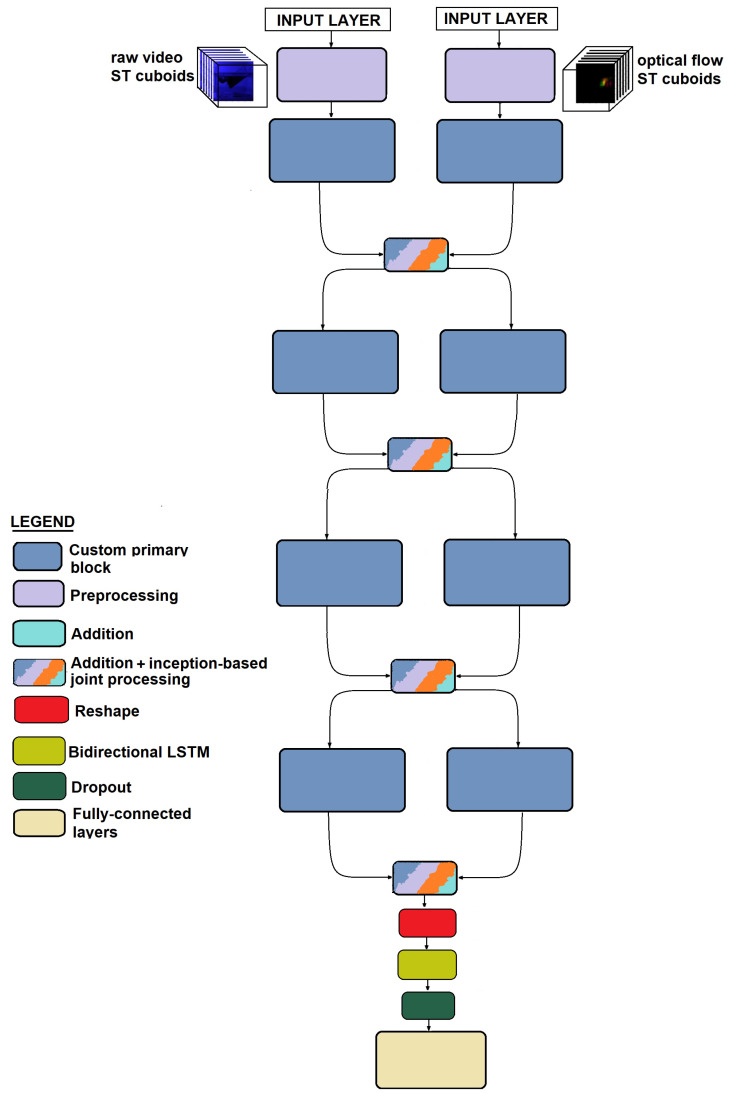
Overview of CIv3D_BiLSTM model.

**Figure A15:**
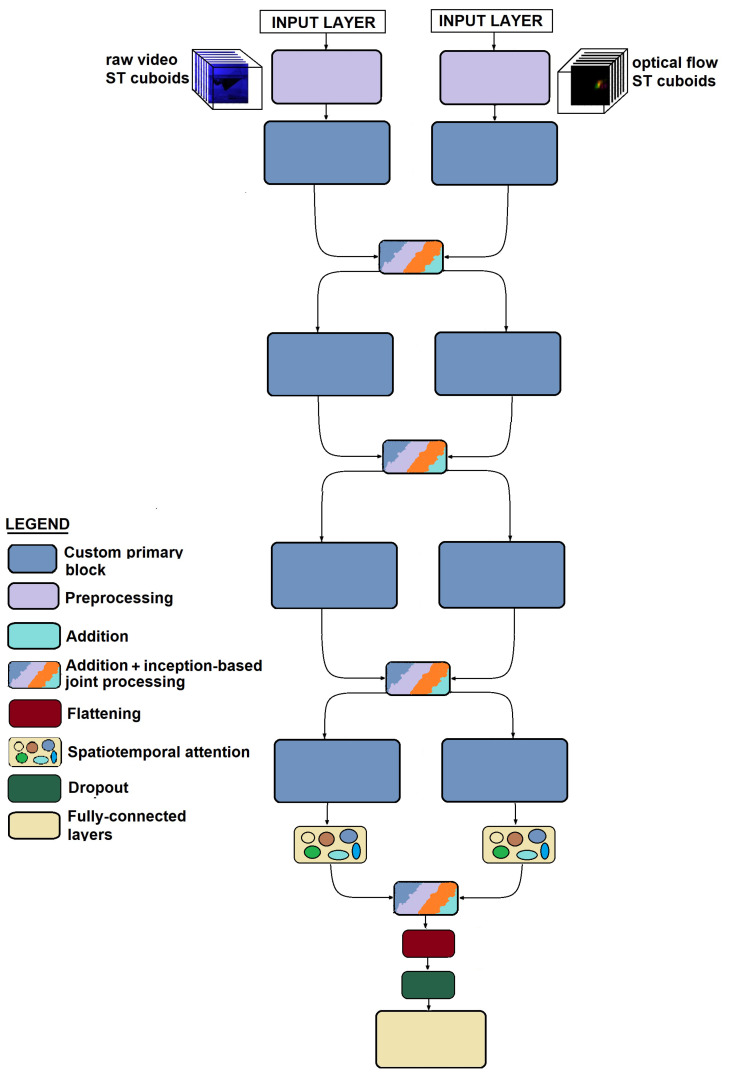
Overview of CIv3D_MHA model.

## Data Availability

All datasets used here are publicly available. The MIT home-cage mouse dataset by [7] is available at https://cbmm.mit.edu/mouse-dataset, (accessed on 20 September 2021). The SCORHE dataset by [10] is currently archived but can be accessed by going to https://web.archive.org, (accessed on 26 July 2022) and then searching for scorhe.nih.gov, (accessed on 26 July 2022). The required videos are available in the “downloads” section.

## Appendix A. Full Performance Table

**Table A1:** Full Performance table for feature sharing models.

MODELS	SEEDS	METRICS				
		AUC		AP	F1 Score	Accuracy (%)
		micro (m)	macro (M)			
Baseline	A	0.89139	0.92472	0.602	0.53434	75.0425
	B	0.87151	0.91915	0.609	0.57256	77.4076
	C	0.86057	0.91478	0.564	0.51816	70.5459
	D	0.89047	0.92020	0.593	0.58093	76.7404
	Average(s)	0.87849	0.91971	0.592	0.55150	74.9341
CIv3D_ BiLSTM	A	0.93746	0. 95331	0.729	0.55750	76.3368
	B	0.92415	0.95030	0.720	0.55634	75.4044
	C	0.92848	0.94623	0.723	0.53677	75.7875
	D	0.91047	0.95133	0.729	0.54228	78.6451
	Average(s)	0.92514	0.95029	0.725	0.54822	76.5435
CIv3D_ MHA	A	0.92600	0.94809	0.692	0.56606	74.2224
	B	0.94715	0.94632	0.757	0.68050	76.2464
	C	0.97321	0.97059	0.860	0.77441	83.6366
	D	0.95560	0.94758	0.772	0.69051	77.8722
	Average(s)	0.95049	0.95315	0.770	0.67787	77.9944
SRS	A	0.96821	0.97703	0.839	0.74434	84.2392
	B	0.95825	0.97383	0.783	0.65857	79.4152
	C	0.94276	0.96119	0.727	0.62004	79.1211
	D	0.96502	0.97416	0.816	0.72210	85.0749
	Average(s)	0.95856	0.97155	0.791	0.68626	81.9626
CRS	A	0.92582	0.95732	0.713	0.56682	77.2607
	B	0.93584	0.96622	0.728	0.57181	79.9323
	C	0.932344	0.95750	0.717	0.56274	78.0969
	D	0.94216	0.95938	0.752	0.60866	78.6730
	Average(s)	0.93404	0.96011	0.728	0.57751	78.4907

## Appendix E. Schematic Diagrams of SRS and CRS Models

The resultant feature shapes of each architecture (at major rungs) and the fully-connected sizes are available in Table 1. As mentioned in the text, the preprocessing steps for all architectures are heightwise cropping and rescaling. The details of the blocks/modules are thus: the *inception-based primary block* in Figure A11 and Figure A12 correspond to Figure 3c, the *custom primary block* in Figure A13, Figure A14 and Figure A15 correspond to Figure 3a, and the *inception-based joint processing* in Figure A14 and Figure A15 correspond to Figure 3b. Furthermore, note that the ST attention block in Figure A15 is the same as a single-head, single encoder stack in [36] but without any positional embedding.
